# Exposure of Cleft Lip and Palate Patients to Toxic Elements Released during Orthodontic Treatment in the Study of Non-Invasive Matrices

**DOI:** 10.1371/journal.pone.0140211

**Published:** 2015-11-06

**Authors:** Marcin Mikulewicz, Krzysztof Kachniarz, Katarzyna Chojnacka

**Affiliations:** 1 Department of Dentofacial Orthopaedics and Orthodontics, Division of Facial Abnormalities, Medical University of Wrocław, Wrocław, Poland; 2 Department of Advanced Material Technologies, Faculty of Chemistry, Wrocław University of Technology, Wrocław, Poland; New York University School of Medicine, UNITED STATES

## Abstract

**The Objective:**

The aim of the study was evaluation of metal ions (nickel and chromium) released from orthodontic appliances in cleft lip and palate patients and the usefulness of non-invasive matrices (saliva and hair).

**Materials and Methods:**

The material studied consisted of 100 individuals, including 59 females and 41 males of 5 to 16 years of age, which were divided into 3 groups: experimental–patients with cleft lip and palate (36 individuals, the average treatment time 5.74 years); control group–patients without cleft lip and palate, during orthodontic treatment (32 individuals, the average treatment time 1.78 years) and the control group patients without cleft lip and palate, without any orthodontic appliances (32 individuals). Samples (saliva, hair) were collected and subjects underwent a survey by questionnaire. Multi-elemental analyses of the composition of non-invasive matrices was conducted in an accredited laboratory by inductively coupled plasma spectrometry technique ICP-OES. The results were reported as mean contents of particular elements (Cd, Cr, Cu, Fe, Mn, Mo, Ni, Si) in hair and in saliva.

**Results:**

The concentration of Cr, Ni, Fe and Cu ions in saliva of cleft lip and palate patients were several times higher as compared with not treated orthodontically control groups and higher than in the group with orthodontic appliances. Among the assessed matrices, hair of cleft lip and palate patients seem to be not a meaningful biomarker.

**Conclusion:**

It was found that orthodontic appliances used in long-term treatment of cleft lip and palate patients do not release toxic levels of Cr and Ni ions.

## Introduction

A cleft lip and palate are a widely spread congenital disorder of the craniofacial area. Approximately 1/700 of the newly-born in the years 1993–1998 [1] are estimated to have been born with the said congenital disorder, with the proportion of females to males being 2 to 1 [2]. Genetic and environmental factors are commonly believed to contribute to the incidence of cleft lip and cleft palate [3].

Therapy administered to cleft lip and palate patients calls for a close cooperation of specialists with the orthodontist. Due to the seriousness of the disorder and required surgery (operations on the palate, the lip; bone grafting, and corrective surgery), orthodontic treatment of such patients requires more time than is the case with non-clefted patients. This in turn elongates the time during which the patient is exposed to the materials of which orthodontic appliances have been made.

The materials used in orthodontics are mainly metal alloys made up of, among others, chromium and nickel. These are held to be allergenic and mutagenic. In the environment of the mouth cavity, these alloys are corrosion-prone, which means that the organism is exposed to a prolonged release of metal ions. The appliances mounted in the oral cavity must meet the ISO and ASTM biocompatibility standards. Biocompatibility is determined by means of *in vivo* and *in vitro* tests. Non-invasive markers of exposure (like hair, saliva, urine, fingernails) are suited for the assessment of the extent of the metal ions accumulation in the organism. These, due to their easy availability and the preciseness of the assessment that they offer, appear to be a useful means for testing for metal ions release into hair and saliva [4–5].

The research into biocompatibility of orthodontic appliances that has been carried out up to now has dealt with a relatively short period of exposure. Different methodologies that were made use of in these studies have yielded varying or inconsistent results. The available reference literature regarding metal ion release from orthodontic appliances does not cover the case of cleft lip, cleft alveolar bone, or cleft palate patients. Hence a study aimed at the assessment of the accumulation metal ions released from orthodontic appliances in the patients undergoing long treatment with non-invasive matrices seems to be called for.

The conditions prevalent in the oral cavity (pH, temperature, microorganisms) facilitate the the process of corrosion. Corrosion products (Ni, Cr and Co ions) are commonly recognized as allergenic and cytotoxic [6]. That is why alloys ought to have good anti-corrosive properties, which means that orthodontic appliances ought to be biocompatible [4]. Reference literature shows that it was hair, saliva, urine and blood that were used as matrices in the *in vivo* studies on the assessment of the ion release from orthodontic appliances.

Saliva and hair, which are non-invasive matrices and are easily available and storable, may be used for the assessment of the biocompatibility of orthodontic alloys in the case of cleft lip, cleft alveolar bone and cleft palate patients. Blood provides hair with nutrients. The chemical elements from blood enter hair tissue, which is reflected in the make-up of the latter. Saliva, on the other hand, is a medium where metals from alloys are being released directly. The difference between the mineral analysis of hair (HMA) and of saliva consists also in a longer detection window [7].

The aim of this paper is to assess the extent of metal ion release from orthodontic appliances in the case of cleft lip and palate patients as well as to evaluate the usefulness of non-invasive matrices for research into the exposure of such patients to metal ions (nickel and chromium) released from orthodontic appliances.

## Materials and Methods

Prior to the studies being carried out, permission from the Bioethics Committee of the Wrocław Medical University in Poland, no. KB-738/2012, was obtained.

### Sampling

The research involved 120 volunteers who expressed their written permission in the form supplied by the Bioethics Commission. All the participants had been instructed as to the aim of the research and its nature.

The study included 100 people who were split into three groups:

1. Experimental group (E): 36 cleft lip and palate patients (hereinafter referred to as **patients with clefts**) undergoing orthodontic treatment. The average treatment time: 5.74 years.
2. Control group 1 (C1): 32 patients free of clefts, undergoing orthodontic treatment. The average treatment time: 1.78 years.
3. Control group 2 (C2): 32 patients free of clefts, not undergoing orthodontic treatment.

The patients undergoing orthodontic treatment (group E and C1) had a fixed orthodontic appliance fitted on both dental arches and made up of: brackets (Victory series; stainless steel, 3M Unitek, Monrovia, California, USA), molar bands (stainless steel, 3M Unitek Monrovia, California, USA), ligatures (stainless steel, 3M Unitek, Monrovia, California, USA) and archwires (stainless steel/NiTi, 3M Unitek, Monrovia, California, USA).

The other control group was composed of healthy patients who were in no need of orthodontic treatment or such treatment had not been begun yet.

### Methodology

The patients had been surveyed before having their non-invasive matrices of hair and saliva sampled.

### Survey methodology

A survey of about 100 questions (including two tables) completed both the patient and the doctor. The queries had been divided into eight groups: information on surgery, general information, hair, etiology, appliances, dietary habits, environment and others. Each survey had an individual number assigned and characteristic of the patient, by means of which also samples of a patient’s hair and saliva were described.

### Hair sampling

Hair samples were collected by the patients themselves in keeping with the instruction they had been provided with, covering the five-month exposure time. The samples were stored in plastic containers prior to being sent to an accredited laboratory where they underwent a multi-elemental analysis.

### Saliva sampling

The patients collected saliva samples by chewing a rubber band till a screw-top plastic container with a capacity of 20 ml had been filled. The sample was frozen prior to being sent to an accredited laboratory, where it underwent a multi-elemental analysis.

### Multi-elemental analysis

Multi-elemental analysis of hair and saliva was performed in the multi-elemental analysis chemical laboratory of the Wrocław University of Technology accredited by the Polish Centre for Accreditation no. AB696 and ILAC—MRA according to PN-EN ISO 17025:2005.

#### Saliva mineralization'

A 25ml sample was subdued to mineralization with 2.5ml 69% m/m of concentrated nitric acid of spectral purity (Suprapur, Merck, Darmstadt, Germany) in Teflon bombs in the Milestone Start D (Shelton, CT, USA) microwave oven. After mineralization of the sample, double de-ionized water purified in the Millipore Simplicity System (Billerica, MA, USA) was added to the sample until the sample reached a weight of 30 g. The digested sample solution then underwent multi-elemental analysis. The concentrations of Cd, Cr, Cu, Fe, Mn, Mo, Ni, Si and Zn were determined by ICP-OES plasma spectrometer with the Varian Vista-MPX (Mulgrave, Victoria Australia) apparatus.

#### Hair mineralization

A 0.5g sample was subjected to mineralization with 69% m/m (5ml) condensed nitric acid of spectral purity (Suprapur, Merck) in Teflon bombs in the Milestone Start D (Shelton, CT, USA) micro oven. After mineralization, doubly de-ionized water, purified in the Millipore Simplicity system, was added to the sample until the sample reached a weight of 50g. The digest solution then underwent multi-elemental analysis. The concentrations of Cd, Cr, Cu, Fe, Mn, Mo, Ni, Si, was marked by ICP-OES plasma spectrometry with the Varian Vista-MPX apparatus.

### Statistical methods

For the purposed of assessing the level of the average levels of particular chemical elements in hair and saliva, basic statistics were calculated: averages (X), standard deviations (SD), standard deviation errors (SDEs). The significance of the groups under scrutiny concerning the average levels of the analyzed chemical elements was assessed by means of one-way analysis of variance ANOVA (F and p-values were given). Scheffé's method was used to evaluate the post-hoc differences between the selected groups of people for each element separately. Scheffé's test takes into account the value of the probability of the test (critical) post-hoc. This test is generally more conservative (ie. gives fewer statistically significant differences between the mean values) as compared with the Newman-Keuls or Duncan test [8]. Differences of p<0.05 were held to be statistically important.

## Results

In Tables 1–4 average values of particular elements in hair and saliva for the three groups under scrutiny have been presented. Statistically significant differences between experimental and control groups were observed for Cu, Fe, Ni and Si in one factor analyses of variations.

**Table 1:** The mean content of elements (mg/kg) in hair of patients from experimental and control groups.

	E, n = 36		C1, n = 32		C2, n = 32		ANOVA		Post-hoc* tests		
	x	SD	x	SD	X	SD	F	*p*	E-C1	C1-C2	E-C2
**Cd**	0.027	0.034	0.037	0.045	0.046	0.074	1.03	0.359	-	-	-
**Cr**	0.012	0.033	0.087	0.156	0.121	0.328	2.53	0.085	-	-	-
**Cu**	14.2	8.84	50.3	101	18.5	12.8	3.7	***0*.*028***	***0*.*044***	-	-
**Fe**	9.15	4.57	11.9	7.1	13.5	9.18	3.16	***0*.*047***	-	-	***0*.*05***
**Mn**	0.393	0.382	0.567	0.519	0.548	0.550	1.34	0.267	-	-	-
**Mo**	0.103	0.102	0.126	0.156	0.097	0.063	0.566	0.570	-	-	-
**Ni**	0.160	0.194	0.643	0.981	0.406	0.653	8.48	***0*.*001***	***0*.*017***	-	-
**Si**	19.9	13.2	63.0	54.2	51.6	57.3	4.29	***0*.*012***	***0*.*001***	-	***0*.*021***

* Scheffe test (p)

**Table 2:** Reference ranges of elements in hair (10^th^-90^th^).

Group	E, n = 36	C1, n = 32	C2, n = 32	Total
**Cd**	0.0000	0.0000	0.0000	0.0000
	0.0652	0.0890	0.1570	0.0890
**Cr**	0.000	0.000	0.000	0.000
	0.059	0.202	0.206	0.175
**Cu**	8.12	10.3	7.70	8.21
	31.8	77.1	39.7	40.5
**Fe**	3.60	6.47	5.75	5.37
	13.7	17.1	26.9	19.8
**Mn**	0.128	0.170	0.093	0.128
	0.922	1.04	1.28	1.06
**Mo**	0.000	0.000	0.000	0.000
	0.191	0.260	0.176	0.191
**Ni**	0.000	0.076	0.053	0.000
	0.466	1.09	0.908	0.748
**Si**	5.94	21.1	15.7	10.8
	34.3	134	122	107

**Table 3:** The mean concentration of elements (mg/kg) in saliva from patients from experimental and control groups.

	E, n = 36		C1, n = 32		C2, n = 32		ANOVA		Post-hoc* tests		
	x	SD	x	SD	X	SD	F	*p*	E-C1	C1-C2	E-C2
**Cd**	0.001	0.002	0.001	0.001	0.001	0.001	0.32	0.726	-	-	-
**Cr**	0.00642	0.019	0.004	0.003	0.003	0.001	0.64	0.53	-	-	-
**Cu**	0.100	0.382	0.057	0.054	0.054	0.034	0.40	0.673	-	-	-
**Fe**	0.346	1.37	0.154	0.088	0.136	0.083	0.65	0.523	-	-	-
**Mn**	0.027	0.084	0.019	0.016	0.017	0.01	0.37	0.692	-	-	-
**Mo**	0.001	0.001	0.001	0.001	0.001	0.001	1.06	0.351	-	-	-
**Ni**	0.00978	0.021	0.008	0.019	0.003	0.002	1.23	0.298	-	-	-
**Si**	5.74	15.6	3.98	6.8	2.07	4.26	0.98	0.379	-	-	-

* Scheffe test (p)

**Table 4:** References ranges of elements in saliva (10^th^-90^th^).

Group	E, n = 36	C1, n = 32	C2, n = 32	Total
**Cd**	0.000000	0.000078	0.000000	0.000000
	0.000485	0.000719	0.000704	0.000606
**Cr**	0.00075	0.00163	0.00211	0.00149
	0.00616	0.00758	0.00512	0.00616
**Cu**	0.00534	0.0126	0.0211	0.00763
	0.107	0.128	0.092	0.107
**Fe**	0.0337	0.0644	0.0693	0.0385
	0.222	0.263	0.229	0.262
**Mn**	0.00334	0.00707	0.00668	0.00445
	0.0505	0.0333	0.0339	0.0343
**Mo**	0.00000	0.00000	0.00000	0.00000
	0.00211	0.00240	0.00245	0.00240
**Ni**	0.00115	0.00000	0.00000	0.00055
	0.0136	0.0141	0.0066	0.0134
**Si**	0.215	0.363	0.125	0.215
	6.87	6.62	4.15	6.09

Data from Tables 1–4 in S1 Data.

Tables 1 and 2 show that for the majority of elements, the level in the group C1 and C2 significantly exceeded the levels in the group E. A statistical significance in the difference of the contents of the elements was noted between groups E and C1 in respect of nickel and silicon, and between groups E and C2 in respect of iron and silicon. It was group C1 that showed the highest content of copper, with the difference between this group and group E being statistically appreciable.

**Fig 1:**
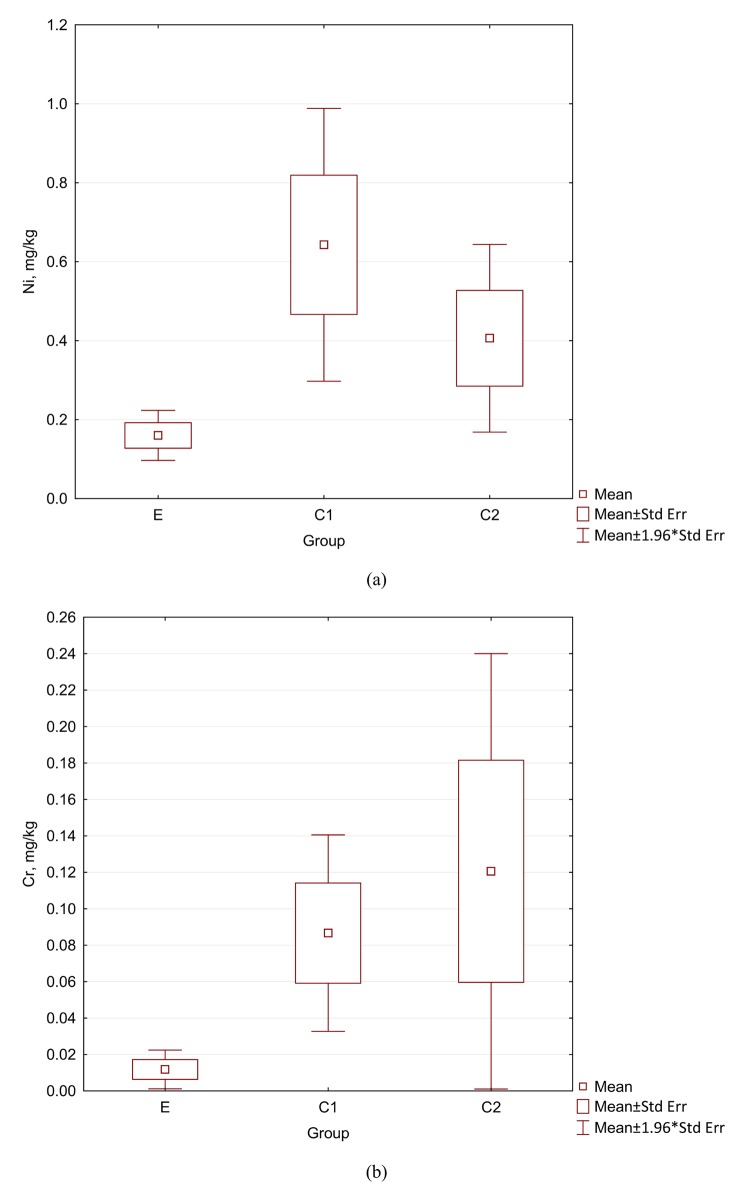
The contents of elements in hair.

The levels of many elements in the group E were visibly lower than in C1 and C2: for Cr (7 times lower for C1 and 10 for C2), Ni (4 and 2.5), Cu (3.5 and 1.3), Si (3.2 and 2.6), Mn (1.4 for both groups), Cd (1.4 and 1.7), Fe (1.3 and 1.5), Mo (1.2 and 0.9). In hair of orthodontic patients without cleft lip and palate (C1)—as compared with subjects not undergoing orthodontic treatment (C2), the release of metal ions to hair tissue was observed, showing that in this case using HMA was meaningful. The differences between C1 and C2 were as follows: for Ni (58% higher in C1 than in C2), for Cu (170%), Mo (30%), Si (22%). The results for Cr and Ni in hair are reported as box-plot graphs in Fig 1A and 1B.

**Fig 2:**
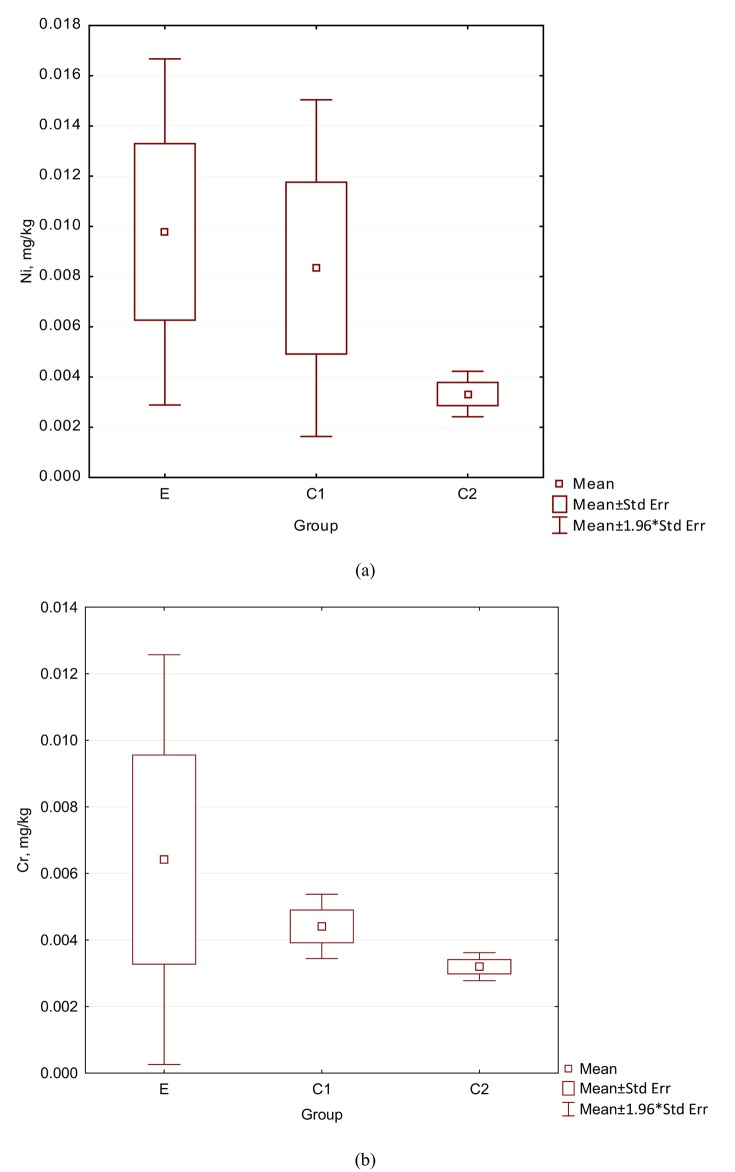
The contents of elements in saliva.

Among the elements occurring in the saliva, there were no significant statistical differences between groups E, C1 and C2. For Cr, the value in saliva in the E group was 0.00642 (mg/kg), followed by lower values in the C1 groups (0.004 mg/kg), followed by 0.003 mg/kg. Although these values were different, and although the value in the E group was higher than those values in the C1 and C2 groups, the differences did not achieve statistical significance (Tables 3 and 4). For copper, the value in saliva in the Group E was 0.1 mg/kg, which was higher than the value in the C1 group (0.057 mg/kg) and higher than the value in the C2 group (0.054) (Tables 3 and 4). However, these differences did not reach statistical significance. For Mn, the value in saliva in the Group E patients, 0.027 kg/kg, was higher than the values in control group 1 (0.019 mg/kg) and higher than the value in control group 2 (0.017, mg/kg). These differences did not reach statistical significance. For the element Ni, the value in group E in saliva of 0.00978 mg/kg, was higher than that in control group 1 (0.008 mg/kg) and higher than that in control group 2 (0.003 mg/kg). However, again, these differences were not statistically significant. The results for Cr and Ni in saliva are reported as box-plot graphs in Fig 2A and 2B.

Data for additional elements in hair and saliva are in S1–S12 Figs.

## Discussion

Among the definitions of biocompatibility that have wide currency is the one that describes the capacity of a material to evoke the desired biological response when applied [9–10]. Biocompatibility is a dynamic process that does not affect the properties of a material but also the interaction of this material with the environment. The evaluation of biocompatibility is not an easy task: it comprises a number of biological tests (*in vitro*, on animals, *in vivo*, usability tests), examination of physical properties, analysis of the market, and the advantages of applying a given material. The biocompatibility standards that have been worked out (ASTM and ISO norms) apart from advantages have also drawbacks [11]. Biocompatibility is in modern stomatology a basic requirement imposed on clinical materials [12]. It is a key requirement that a relationship between the contents of toxic elements (like nickel ions released from orthodontic appliances) in tissues and their negative influence on health be determined [13]. Usually blood and urine are selected for this purpose. These are very-well known biomarkers that provide creditable results. Urine analysis is often made use of in diagnosing various ailments. In the case of acute intoxication the detection window for urine amounts to 36–72 hours [5]. Human hair is yet another biomarker: it is easily available and storable, and does not need to be preserved. A hair grows approximately 1cm a month [14]. Sampling a hair at different distances from the skin allows for the selection of exposure time window. Mineral analysis of hair is recognized as a significant marker of a prolonged exposure to metals, among them to nickel, and the results correspond to blood sample results [15–19]. Mineral analysis of hair is recommended by US Environmental Protection Agency (U.S. EPA) and International Atomic Energy Agency (IAEA) to be used as one of the most credible biomarkers of exposure to metals [20].

On the basis of mineral analysis of hair the researchers have proved that an average two-year orthodontic treatment with the application of permanent appliances exposes to nickel. The results of the recent study [21] thanks to more advanced measuring tools are more precise than the previous ones [22], where the contents of nickel ions in hair is smaller in the group of patients undergoing orthodontic treatment than in the control group. No increase in the amount of nickel in the hair of the patients undergoing orthodontic treatment is accounted for by means of a much smaller absorption of nickel ions released in the mouth than of those supplied with the nutrients and connected with a particular diet. In a study by Mikulewicz et al. [7] samples had been taken prior to treatment, within four, eight and twelve weeks of mounting the orthodontic appliances. The researchers present a statistically significant increase in the amount of Cr during treatment as comparted to the time prior to it.

In this paper, mineral analysis of hair was selected as a biomarker for the assessment of the exposure of the patients undergoing orthodontic treatment to toxic metals. The obtained results overlap with earlier studies where nickel ions were bioavailable through different matrices, such as urine, blood and saliva [16, 19, 23–25]. Reference literature shows a lack of long-term *in vivo* research that might assess prolonged exposure to metals during orthodontic treatment among healthy patients [22] or any concerning patients with clefts.

The average contents of Cr in none of the groups E– 0.012 mg/kg, C1–0.087 mg/kg, C2–0.121 mg/kg exceeded the upper norm limit for particular reference ranges laid down. It was a patient from group C1 who had the highest amount of Cr 0.82 mg/kg in hair, which did not exceed the norm that was found out by Chojnacka et al. [26] for the students of Wrocław University of Technology, which is 1.53 mg/kg. The highest average amount of Cr occurred in group C2, and the lowest in group E. The average amounts of Cr in the study by Mikulewicz et al. (2014) amounted to 0.0201 mg/kg for patients prior to orthodontic treatment corresponding to group C2 in this study, and to 0.158 mg/kg among the patients after a twelve-week exposure to orthodontic appliances, which is comparable to group C1. All the results quoted by Mikulewicz et al. (2014) were higher than for group E presented herein. This higher contents of Cr in Control group C2 than in the control group from the quoted study can be accounted for by a different domicile of the individuals under study (Wrocław vs Kraków), among others by air pollution and different composition of drinking water [27].

It was group E, where the lowest contents of Ni was noted—E– 0.160 mg/kg—and it was group C1, where this content was the highest: 0.643 mg/kg. The difference was statistically significant. The values of group E were also lower than those of group C2: 0.406 mg/kg. The average amounts of Ni in the three groups did not exceed the upper norm limit for the reference ranges found out by Rao et al. [28], Chojnacka et al. [26] and Dongarra et al. [29]. The average Ni content in the study by Mikulewicz et al. [7] amounted to 0.288 mg/kg for the individuals prior to the commencement of orthodontic treatment, which corresponds to group C2 in this paper, and 0.422 mg/kg among the patients after a twelve-week exposure to metal ions released from orthodontic appliances, which can be compared to group C1. All the results for Ni presented by Mikulewicz et al. [7] were higher than the average values in group E of this study.

Apart from the average Cu contents in group C1, the average contents of the elements in each of the groups did not exceed the maximum values, which confirms earlier conclusions [7, 22] that the exposure to metal ions from orthodontic appliances cannot be hazardous for health.

The average contents of all the elements under study among the patients with clefts undergoing prolonged treatment was smaller as compared to healthy individuals whose exposition to ion release from orthodontic appliances was much shorter. This can be accounted for by a possible anomaly of the absorption of minerals or by different hair structure [30–31]. The contents of the element in hair overlaps with a number of diseases and the contents is often lower than among healthy individuals. The Fe level lower in Parkinson’s disease [32], the Cu level lower in autistic children [33] and children with rheumatism [34], Mg, Fe, Cu, Mn lower among women suffering from breast cancer [35].

The results of the present study overlap with those of the previous ones, like those by Agaoglu et al. [23], who observed an increase in the Cr and Ni concentration in saliva within a year after orthodontic appliances had been mounted. Fors&Persson [36] showed that the amount of Ni in dental plaque retained by filters was much higher in patients undergoing orthodontic treatment as compared to the control group within an average sixteen-month time of saliva sampling.

On the basis of the difference between the ion concentration of Cr and Ni in the study and control group the exposure of the patients with clefts to toxic doses of these elements was assessed, according to the following equation:

$$r_{element}\left(mg/d\right)=\left(C_{element}^{E}-C_{element}^{C2}\right)\left(mg/L\right)\cdot {\dot{V}}_{saliva}\left(L/d\right)$$

where:

*r_element_*(*mg/d*)—rate of metal ions release

*C_element_*(*mg/L*—concentration of metal ions in saliva in a given Group

${\dot{V}}_{saliva}\left(L/d\right)$—flow rate of saliva (assumed as 1.5 L/d).

Within twenty-four hours the orthodontic appliances of the patients with clefts would release 4.60 μg/d of Cr and 5.52 μg/d mg of Ni ions. In light of the daily demand as determined according to WHO norms (50–200μg/d Cr, 25–35μg/d) the released ions make up 9.2% (Cr) and 22.1% (Ni) of the daily demand [37]. Which means, by way of example, that if treatment of a patient with a cleft lasts 10 years, the amount of the released ions will have reached 16,800μg of Cr and 20,150μg of Ni by the end of it.

In the case of orthodontic patients without clefts, 1.02μg/d ions of Cr and 4.42 μg/d ions of Ni are released, which makes up 2.04% and 17.7% respectively of the daily demand. Reference literature makes known the results of the element analysis of the saliva from the patients undergoing orthodontic treatment. In the studies concerned with samples of saliva the concentration of Ni ranged between 0.49ng/ml [38] and 78ng/ml in the experimental group [39], and between 1.16ng/ml [38] and 34ng/ml [39] in the control group, with the average concentration of Ni in the three adduced papers being higher in the control group [23, 38, 40] as compared to the experimental one. Kocaderelli et al. [38] have found out that there were no statistically significant differences in the amount of Cr and Ni in saliva prior to and after the mounting of orthodontic appliances. Eliades et al. [40] has not found out the amount of the released metal ions to be higher than the daily dose absorbed with nutrients or with the inhaled air. Agaoglu et al. [23] note that Ni and Cr ions are released from orthodontic appliances in different amounts in proportion to the time of their presence in the oral cavity, but still their levels in saliva do not approach toxic values but are close to the levels in individuals without appliances. The results presented in the papers where the levels of the released metal ions were higher in the experimental group than in the control group [25, 41, 42] are also statistically insignificant. Amini et al. [24] note that in patients under orthodontic treatment the nickel levels in saliva do not reach toxic values. A low level of these ions may pose a threat to allergic patients. Eliades et al. [40] and Gjerdet et al. [43] have not observed a higher level of metal ion contents in patients under orthodontic treatment. Still other studies were carried out either within a short spell of time between 1 week and 3 months [38, 39, 44] or they were *in vitro* studies. The obtained results of metal ion concentrations in saliva cannot be compared with the available studies due to the absence of the group consisting of patients with clefts. Metal corrosion and Ni or Cr ion release do not translate into a linear dependence on time [40]. Except for a number of reports [23, 36, 40], in the majority of earlier publications the number of ions in the oral cavity was counted within a very short period (1–3 months). Such a short period is not sufficient for an effective assessment of metal ions concentration in saliva of orthodontic patients [40]. Only the study by Amini et al. [24] aimed at a 12–18-month treatment.

Most orthodontic appliances are made of alloys of stainless steel and NiTi alloys [45] that can release ions into the oral cavity [46–48]. Corrosion of orthodontic appliances and metal ion release in the environment of the oral cavity are recognized as two main factors for metal ion release. One of them is manufacturing that has to do with the type of alloy and the properties of its constituent metals [49]. The other one is the environmental conditions, diet, time of day, saliva flow rate and psychosomatic condition of a given patient as well as mechanical stress [43, 50].

Another acid factor is the condition of the oral cavity, products containing fluoride such as mouth wash and tooth paste [51–52].

## Conclusions

1. Metal ions from orthodontic appliances in patients with a cleft lip, alveolar bone cleft and a palate cleft are released in doses that are far below the toxic level.
2. Among the non-invasive matrices (hair, saliva), saliva appears to be a creditable biomarker of the exposure to metal ions released from orthodontic appliances.
3. Statistically insignificant differences in the contents of nickel in the hair of orthodontic patients without clefts and of those from the control group have been discovered. The concentrations of chromium, nickel, iron and copper ions in the saliva of the patients with clefts were a few times as high as in the control group undergoing no treatment and appreciably higher as compared to the control group undergoing orthodontic treatment.
4. The element composition of the hair of the patients with clefts differed from the element composition from control groups (undergoing and not undergoing orthodontic treatment).
5. Chromium and nickel ions undergo solubilization from orthodontic appliances worn by patients with clefts (visible differences in saliva composition); however, ions appear not to be released into hair tissue.
6. Two additional factors affecting the level of chromium and nickel in hair (additional exposure) have been found: silver and gold jewelry as a source of nickel, and plumbing (release of copper ions from the plumbing made of its alloys). Hair care also affects the elemental composition of hair (drying).
7. Comparative analysis of saliva composition in the patients with clefts and that of the patients not undergoing orthodontic treatment has shown that chromium and nickel ions are not released in toxic does during treatment.

## Supporting Information

S1 Data Data from Tables 1–4. (XLSX)

S1 Fig The content of Cu in hair. (TIFF)

S2 Fig The content of Fe in hair. (TIFF)

S3 Fig The content of Si in hair. (TIFF)

S4 Fig The content of Cd in hair. (TIFF)

S5 Fig The content of Mn in hair. (TIFF)

S6 Fig The content of Mo in hair. (TIFF)

S7 Fig The content of Cu in saliva. (TIFF)

S8 Fig The content of Fe in saliva. (TIFF)

S9 Fig The content of Si in saliva. (TIFF)

S10 Fig The content of Cd in saliva. (TIFF)

S11 Fig The content of Mn in saliva. (TIFF)

S12 Fig The content of Mo in saliva. (TIFF)
