# Supplementary figures and images for: Exposure of Cleft Lip and Palate Patients to Toxic Elements Released during Orthodontic Treatment in the Study of Non-Invasive Matrices
Source: PLoS One. 2015 Nov 6;10(11):e0140211. doi: 10.1371/journal.pone.0140211 (PMC4636263; doi:10.1371/journal.pone.0140211)

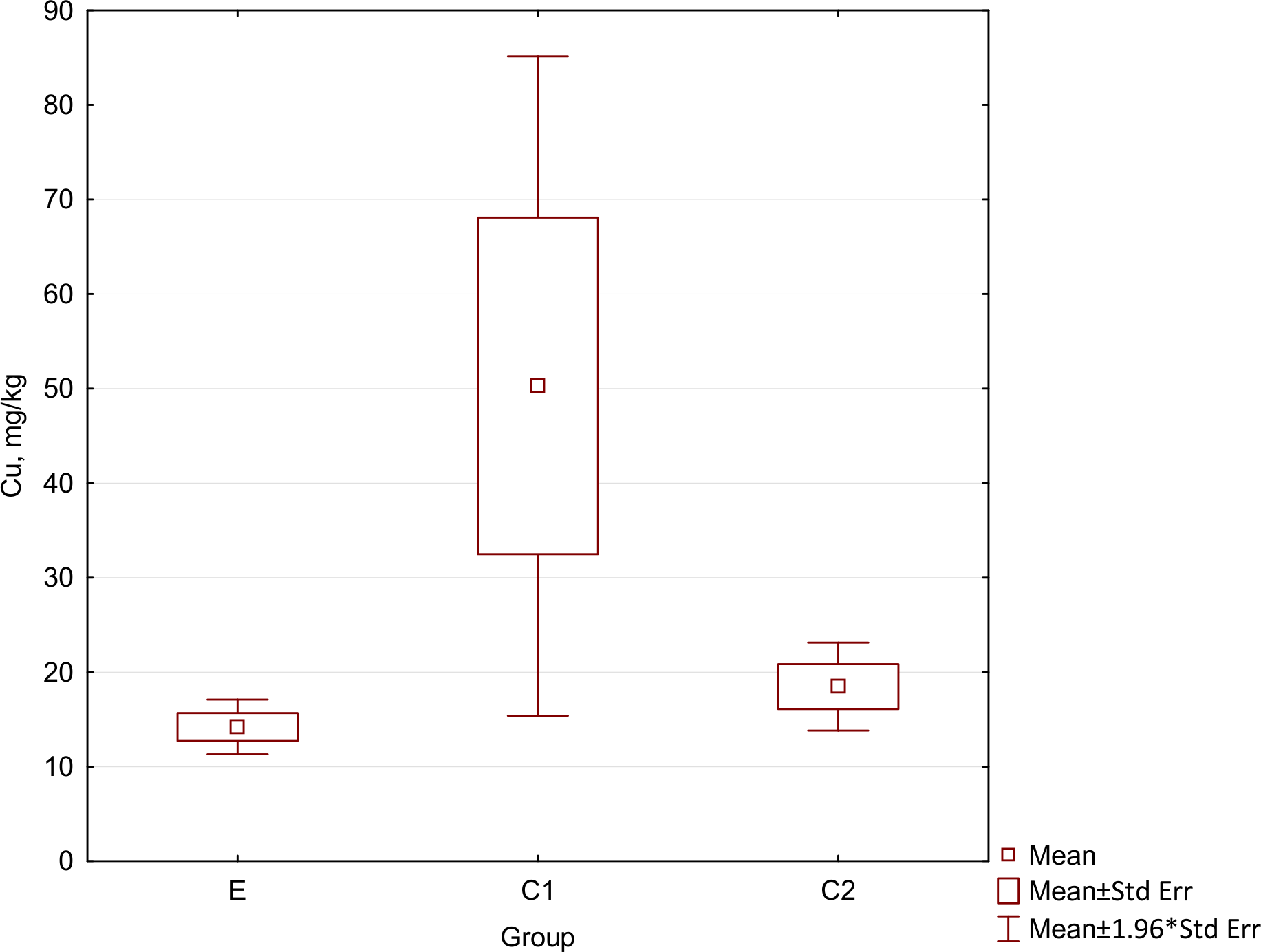

Supplement: S1 Fig — (TIFF) [file pone.0140211.s002.tiff]

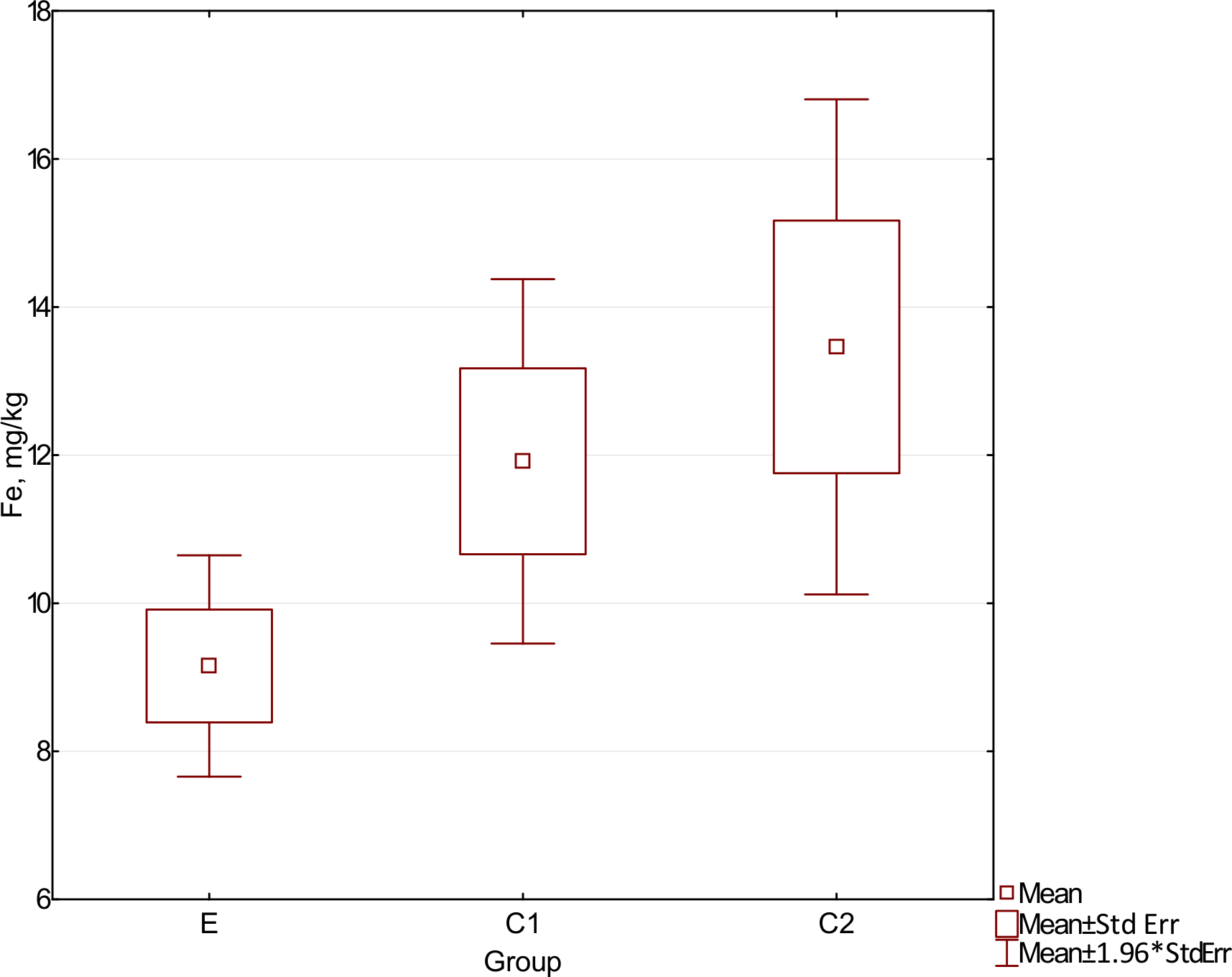

Supplement: S2 Fig — (TIFF) [file pone.0140211.s003.tiff]

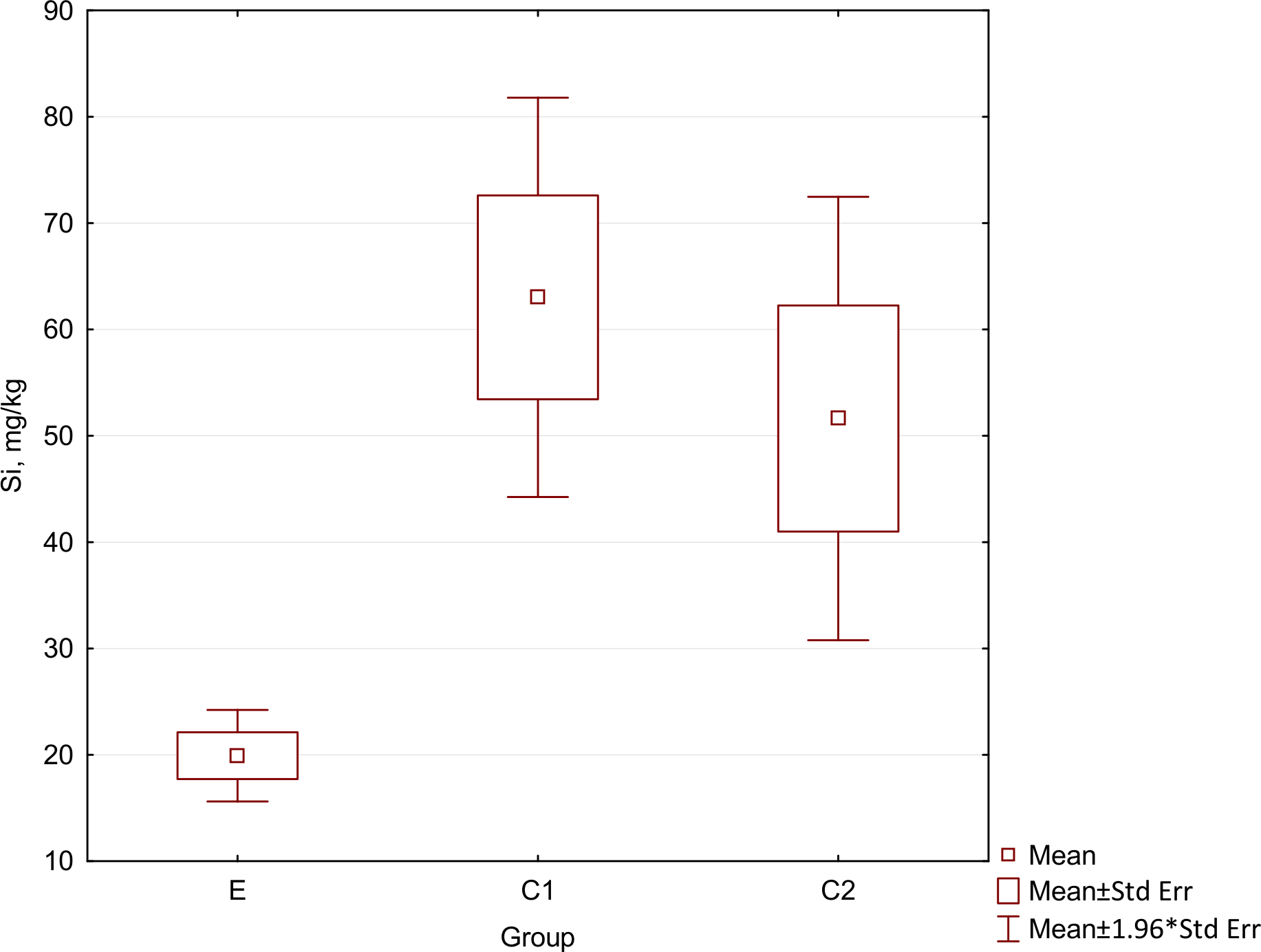

Supplement: S3 Fig — (TIFF) [file pone.0140211.s004.tiff]

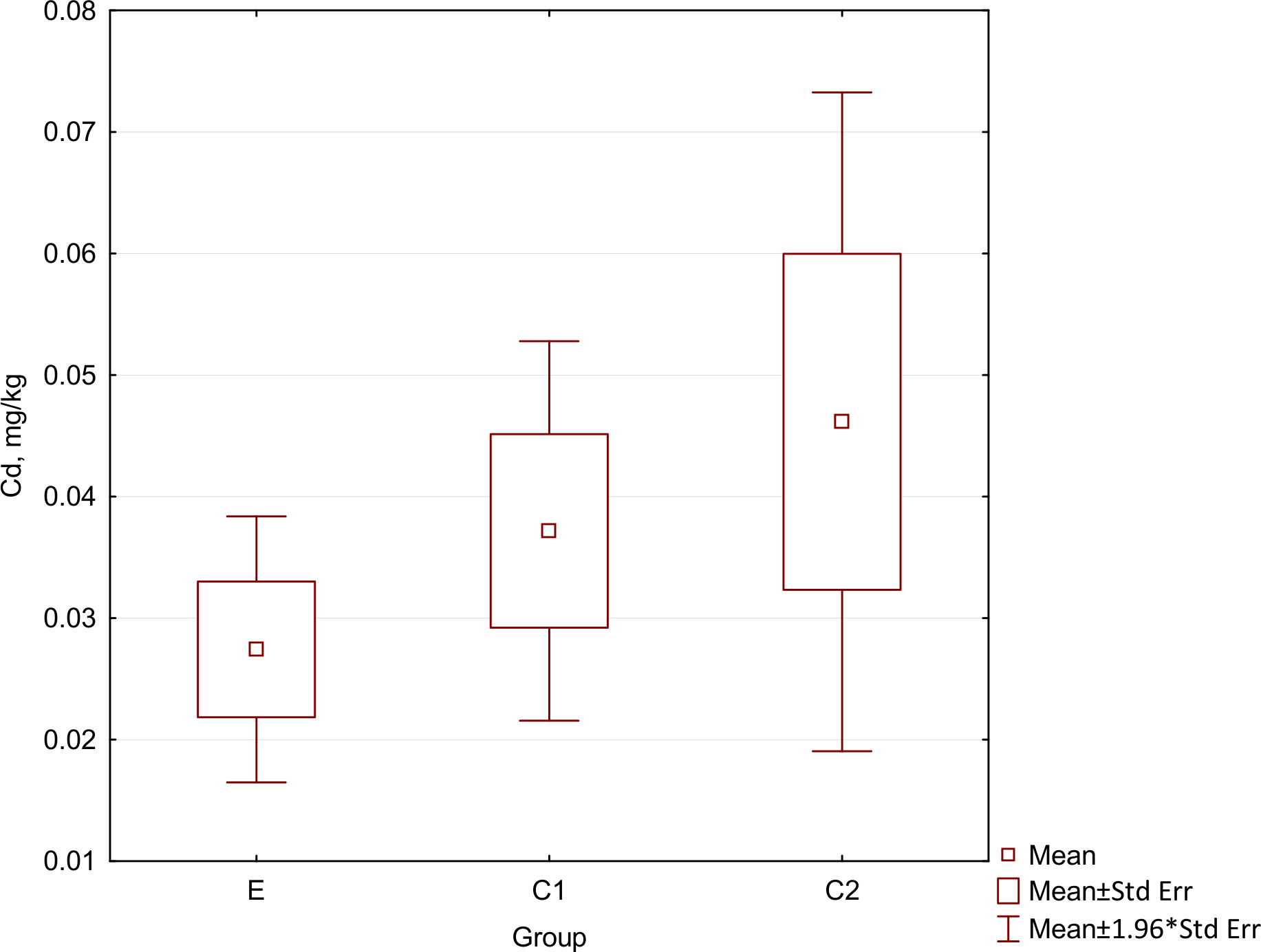

Supplement: S4 Fig — (TIFF) [file pone.0140211.s005.tiff]

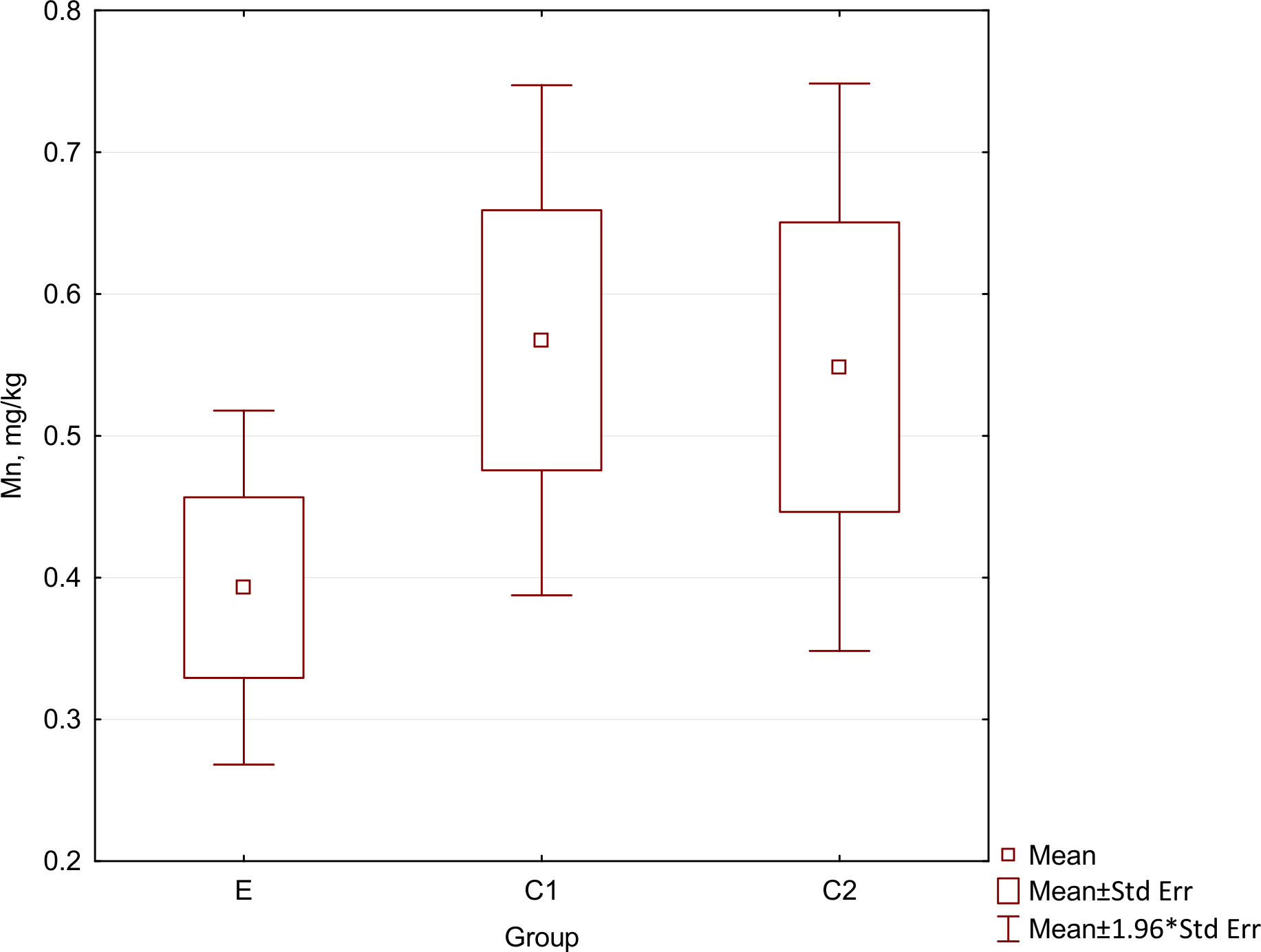

Supplement: S5 Fig — (TIFF) [file pone.0140211.s006.tiff]

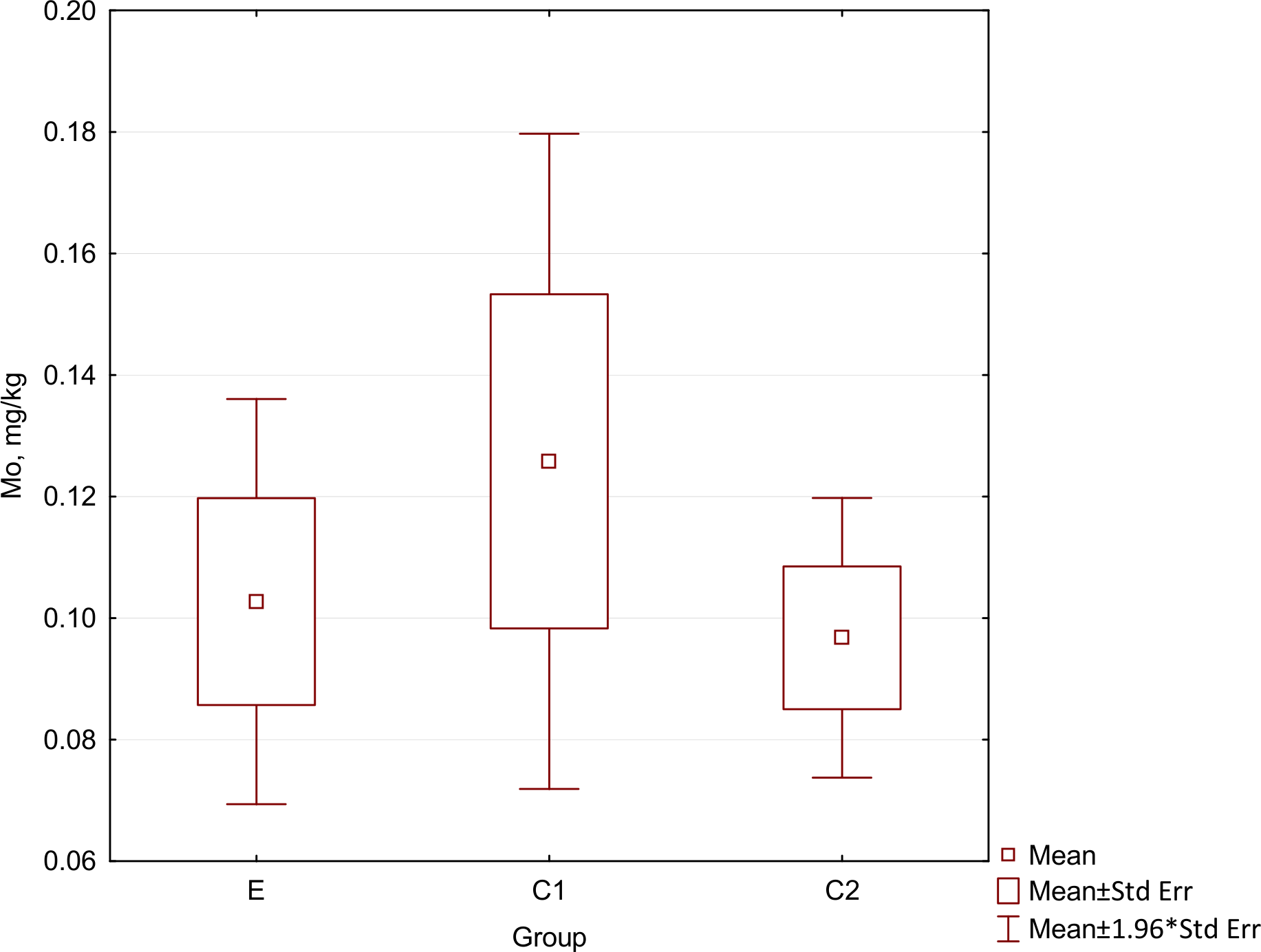

Supplement: S6 Fig — (TIFF) [file pone.0140211.s007.tiff]

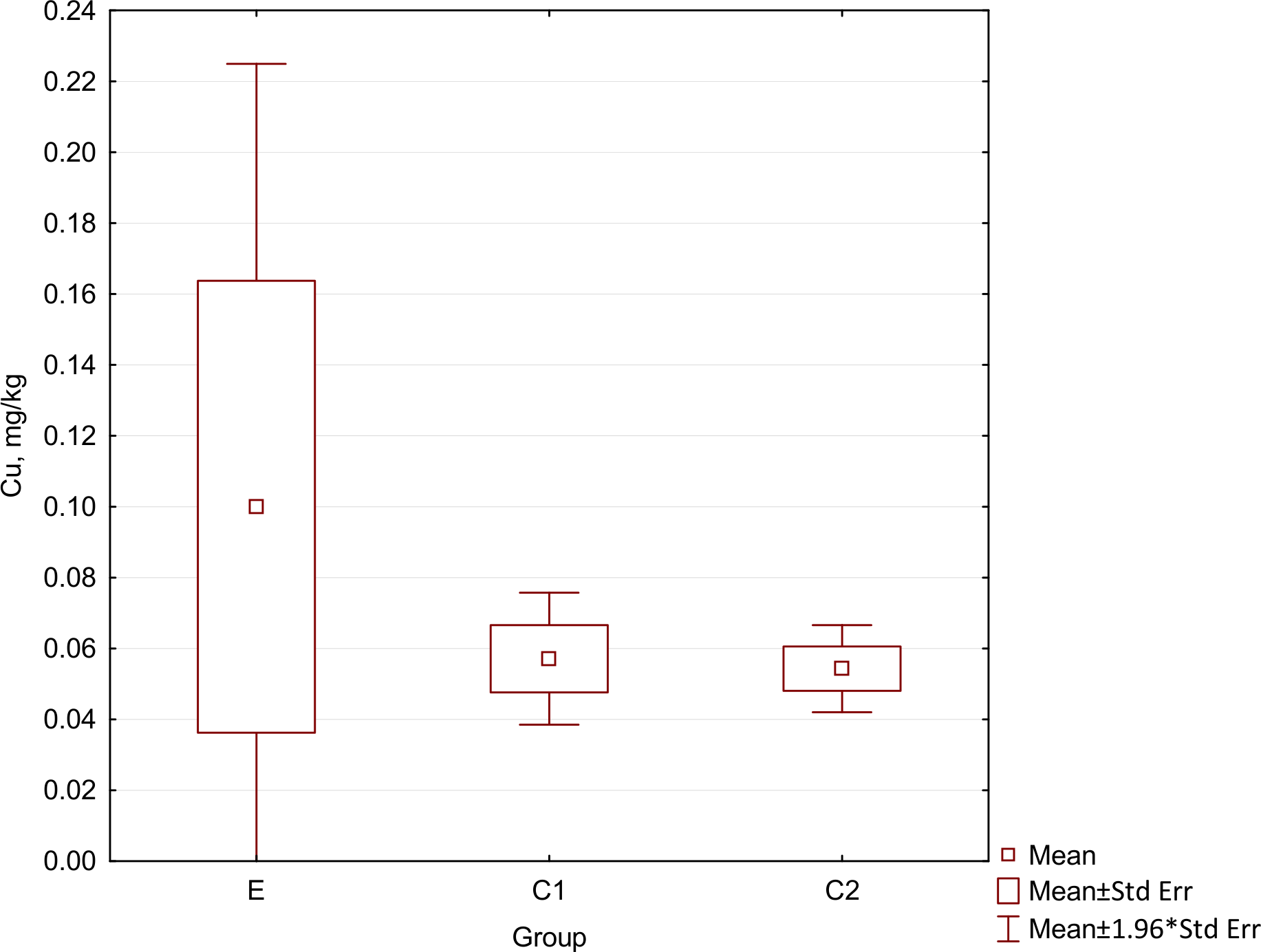

Supplement: S7 Fig — (TIFF) [file pone.0140211.s008.tiff]

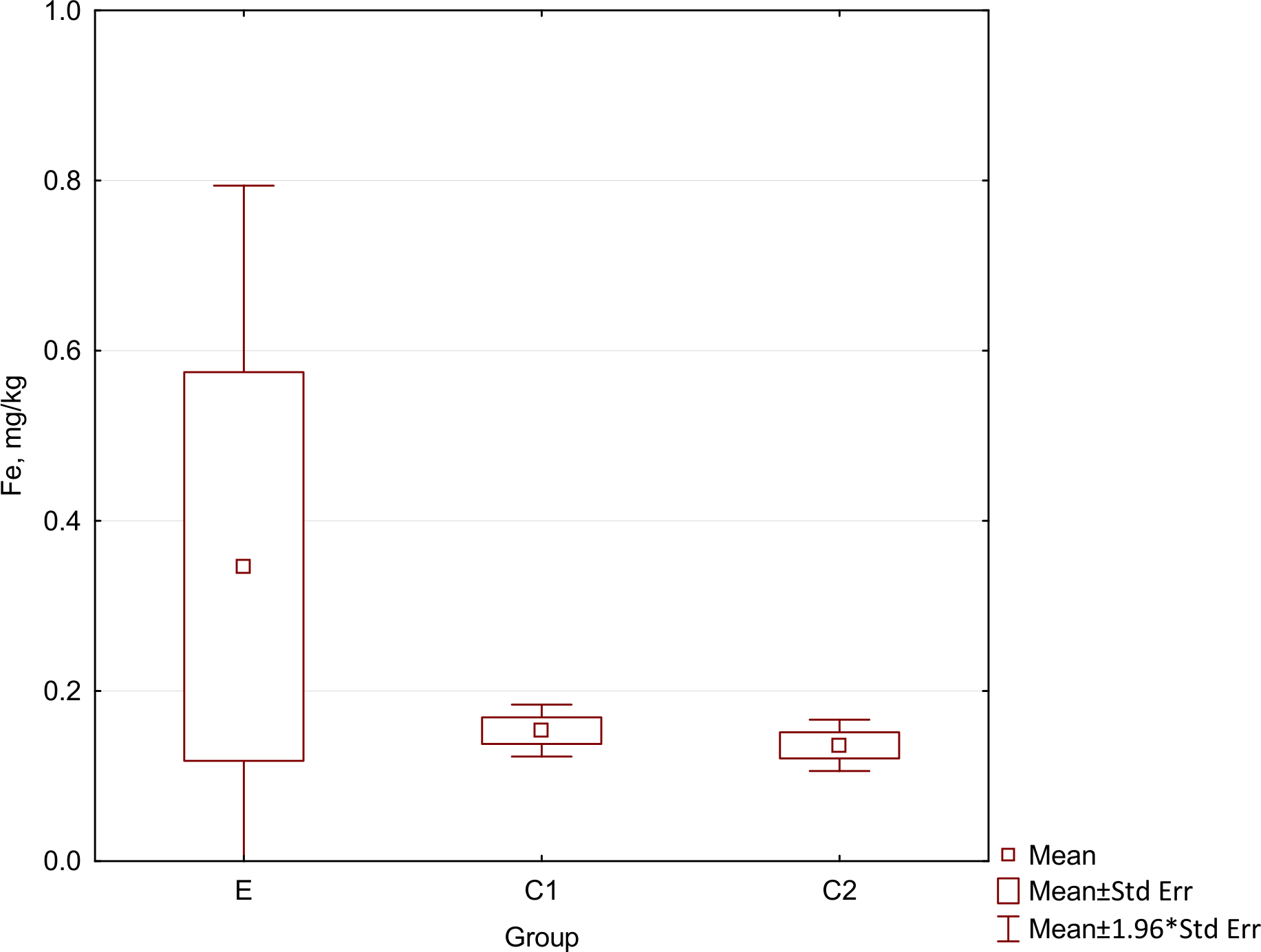

Supplement: S8 Fig — (TIFF) [file pone.0140211.s009.tiff]

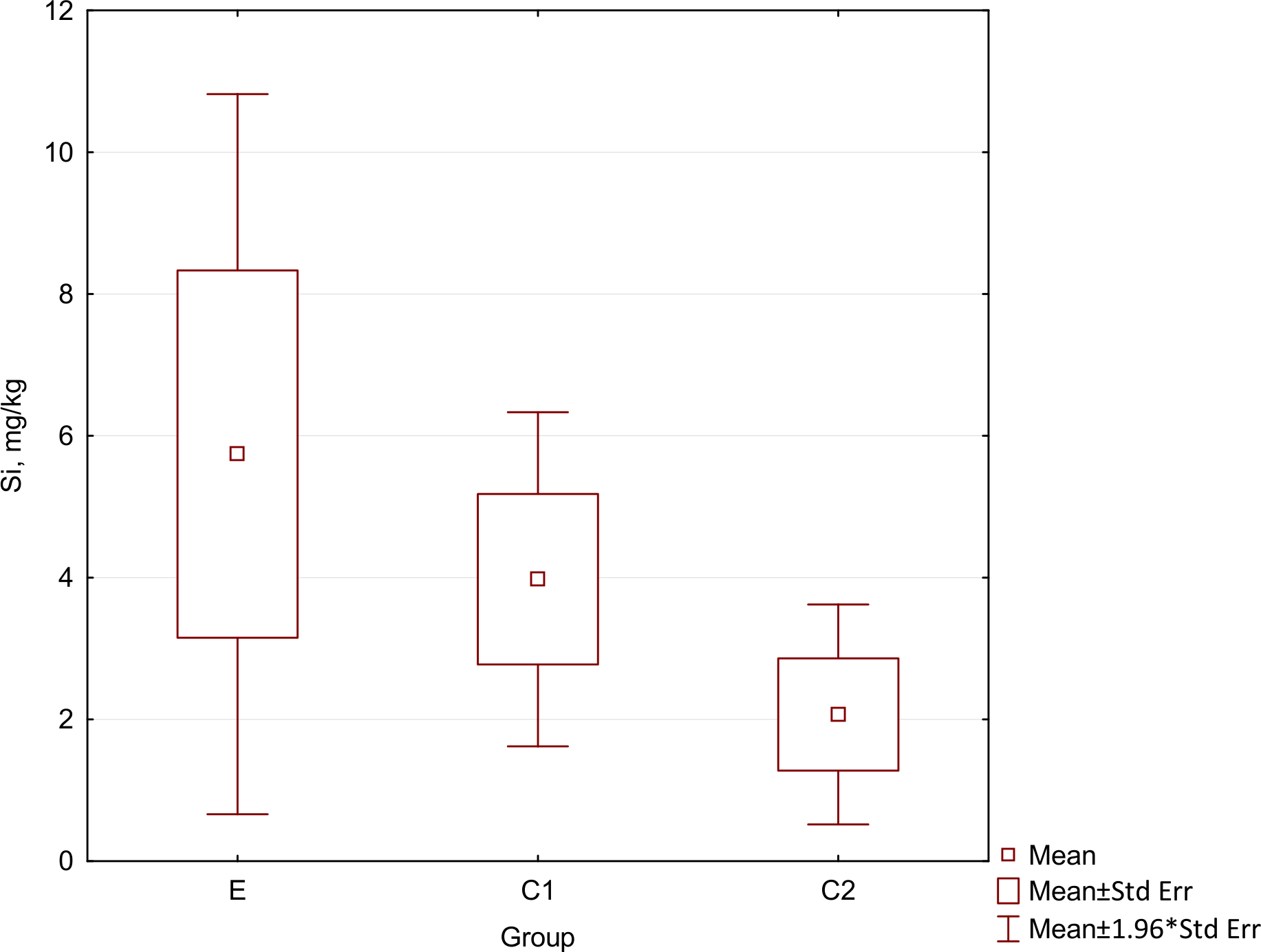

Supplement: S9 Fig — (TIFF) [file pone.0140211.s010.tiff]

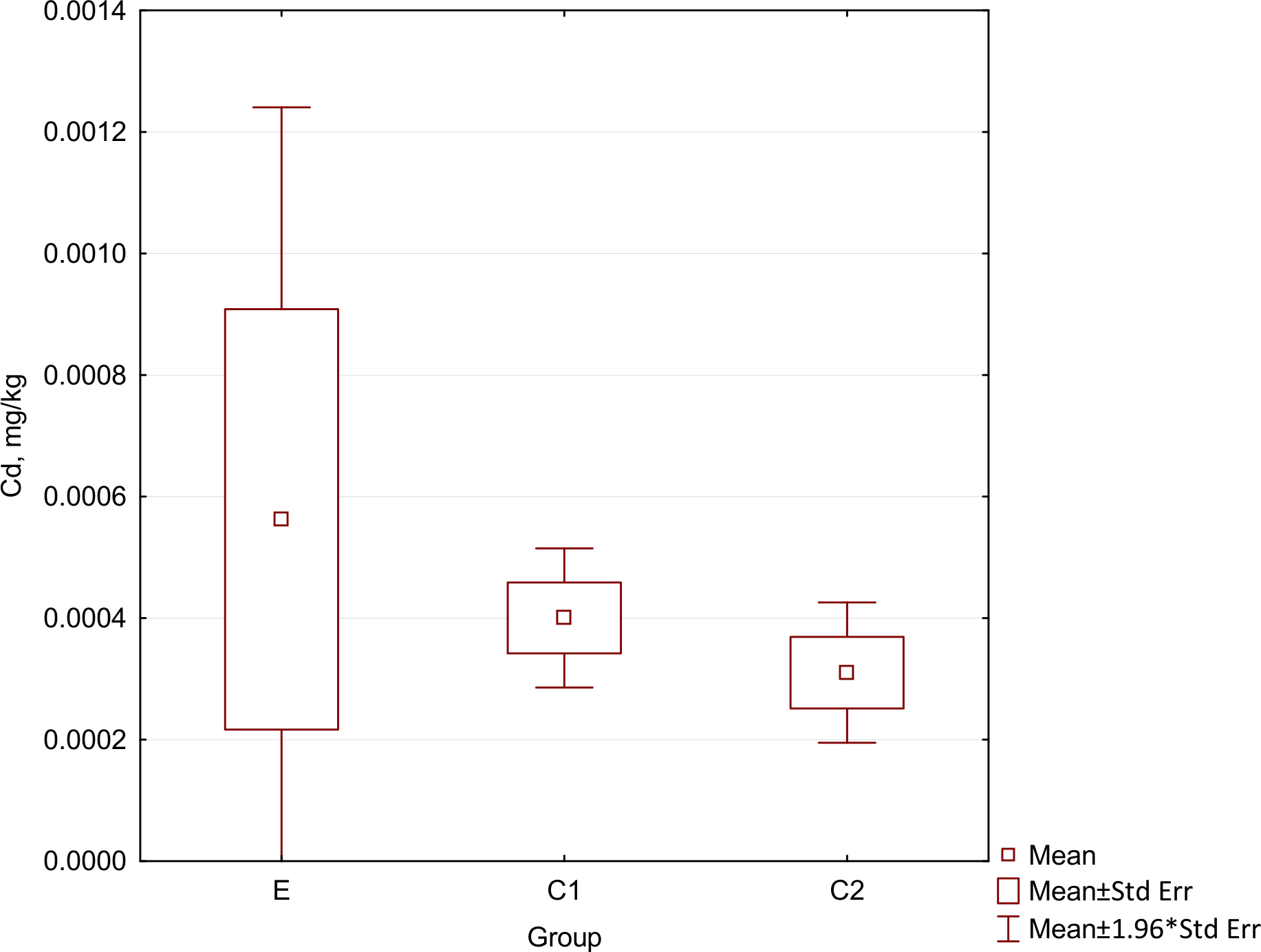

Supplement: S10 Fig — (TIFF) [file pone.0140211.s011.tiff]

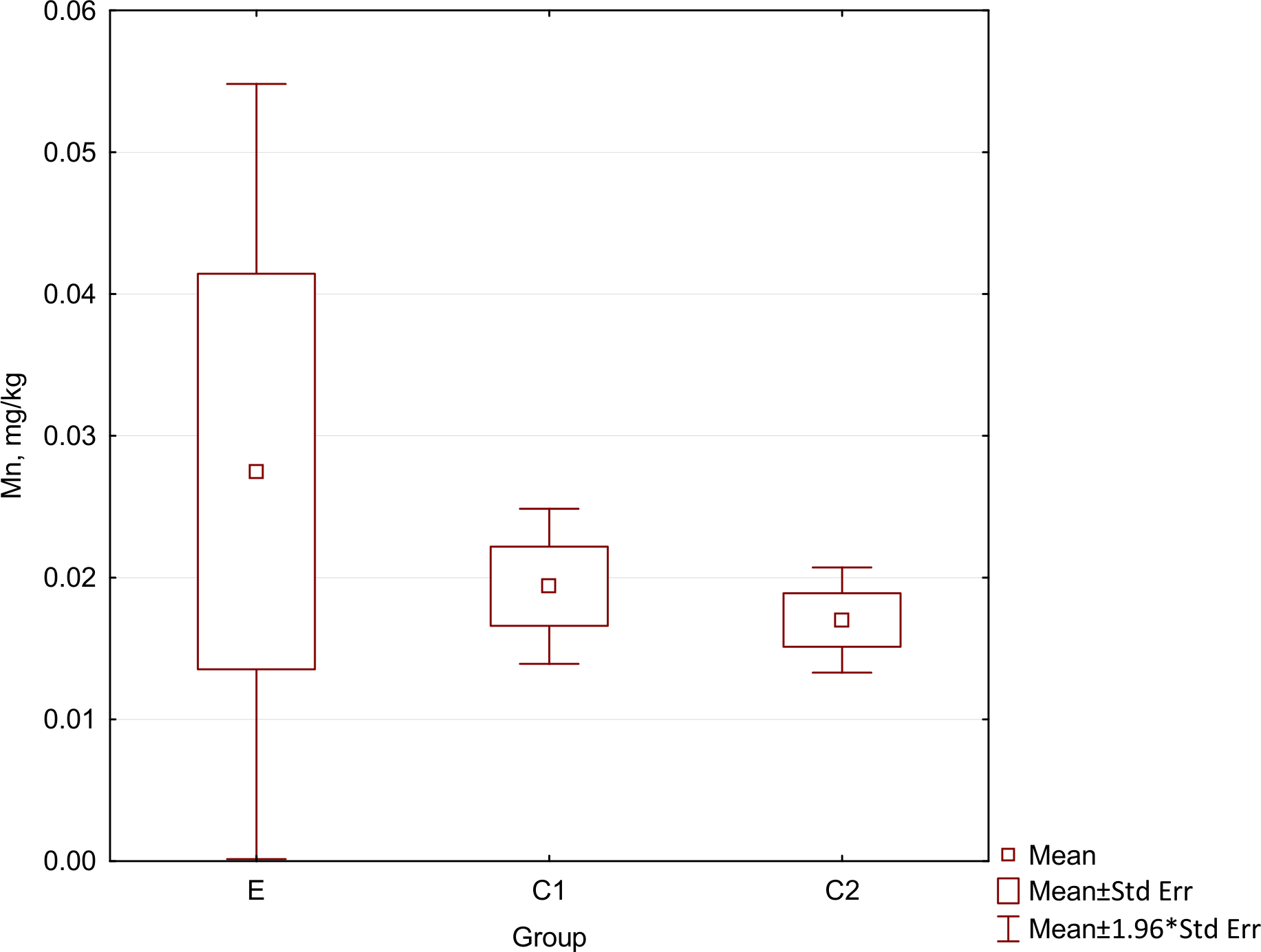

Supplement: S11 Fig — (TIFF) [file pone.0140211.s012.tiff]

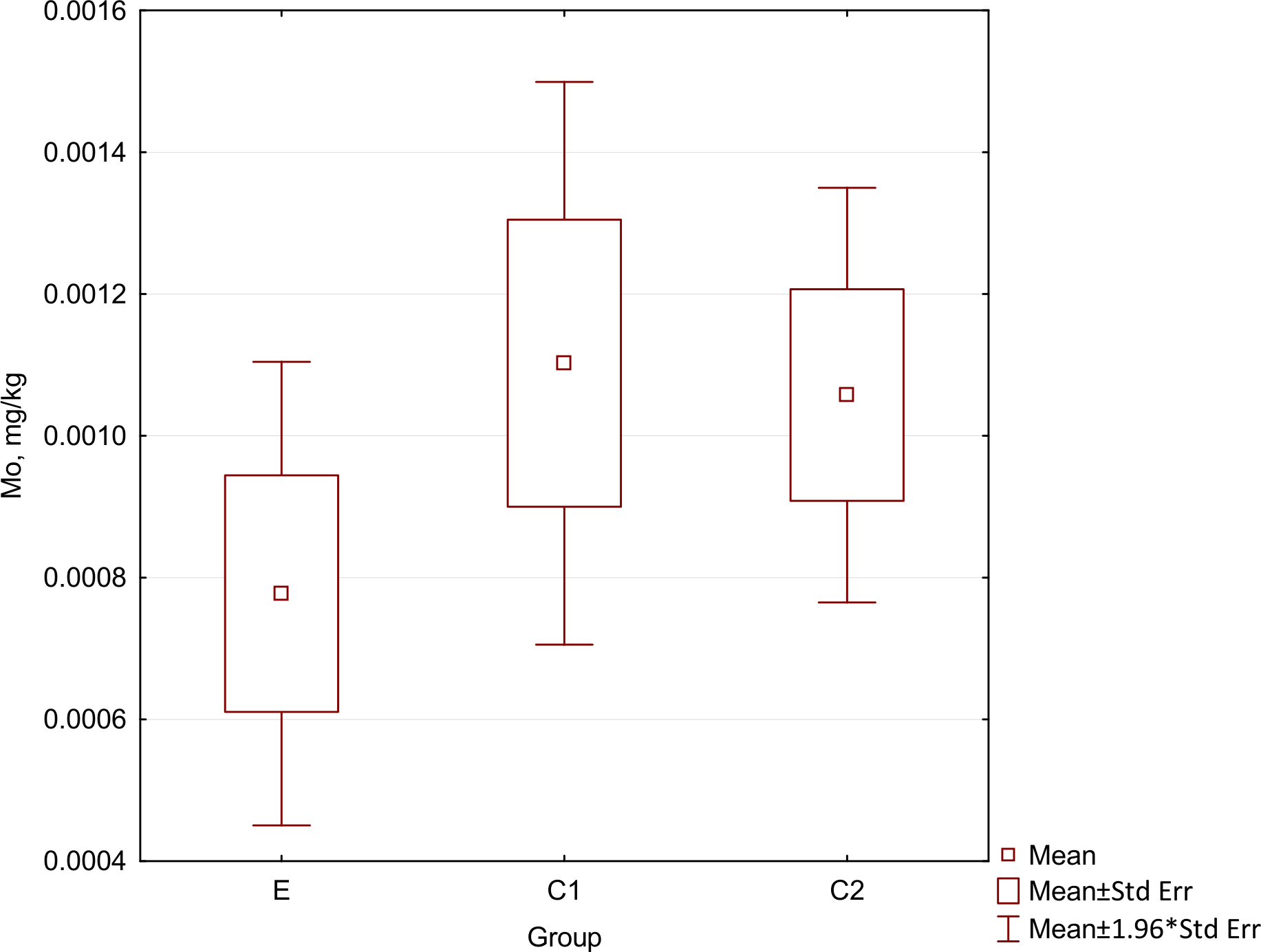

Supplement: S12 Fig — (TIFF) [file pone.0140211.s013.tiff]
